# Cryo-EM structure of shutdown human nonmuscle myosin 2A

**DOI:** 10.1126/sciadv.aed1858

**Published:** 2026-04-17

**Authors:** David Casas-Mao, Glenn Carrington, Michelle Peckham

**Affiliations:** Astbury Centre for Structural Molecular Biology & School of Molecular and Cellular Biology, Faculty of Biological Sciences, University of Leeds, Leeds, UK.

## Abstract

Determining the high-resolution structure of the widely expressed nonmuscle myosin 2A (NM2A), in its dephosphorylated shutdown state, is important in understanding its regulation and disease roles. In shutdown molecules, the coiled-coil tail wraps around the myosin heads, preventing them from forming filaments and binding to actin. We have solved the shutdown structure of NM2A to a global resolution of 3.0 angstroms in the head region and 6.3 angstroms for the whole molecule. This reveals specific ionic interactions that explain why the path of the coiled coil and the shutdown mechanism for NM2A differ from those of β-cardiac myosin and provides key insight into how specific mutations likely destabilize the shutdown state, leading to disease.

## Teaser

The structural basis for the shutdown state of the platelet myosin, nonmuscle myosin 2A.

## INTRODUCTION

Myosins, molecular motors that move along filamentous actin (F-actin) driven by adenosine 5′-triphosphate (ATP) hydrolysis, are composed of a complex of heavy chains (HCs) and light chains. The N-terminal region of the HC comprises the motor domain, which contains both nucleotide and F-actin binding sites. The motor is followed by the lever that, in all class 2 myosins, comprises the converter domain, followed by an α helix containing two IQ motifs, to which one essential (ELC) and one regulatory (RLC) light chain bind (*1*). The lever is followed by a long stretch of coiled coil in class 2 myosins, which both dimerizes the two HCs and enables filament assembly, through its repeating patterns of charged residues along its length (*2*).

Smooth and nonmuscle class 2 myosins switch between an active filamentous state and individual shutdown (10*S*) molecules (*3*–*5*). In the shutdown state, the coiled-coil tail wraps completely around the heads, and the adenosine triphosphatase of these molecules is very low (*6*). Phosphorylation of the RLC, primarily at Ser19 (S19) by myosin light chain kinase (MLCK) (*7*–*9*), disrupts the shutdown state, allowing myosin to extend and then assemble into filaments [reviewed in (*10*, *11*)].

Relatively high-resolution structures for shutdown smooth muscle myosin (SMM) (up to 3.4 Å) in the myosin head region have been reported (*12*–*14*) and used to build a homology model for nonmuscle myosin 2A (NM2A) (*15*). However, a high-resolution structure for shutdown NM2A has not yet been reported. NM2A is the only isoform of class 2 myosin found in platelets (*16*). On platelet activation, RLC phosphorylation rapidly increases from ~10% (*17*) to about 90% within ~1 min (*18*), driving filament formation and platelet spreading, demonstrating the critical role of RLC phosphorylation in the biological function of NM2A. Critically, more than ~200 autosomal dominant missense mutations have been reported for the myosin heavy chain 9 gene (MYH9) that encodes NM2A HC, which cause bleeding disorders, kidney abnormalities, hearing defects, and other disorders (*19*–*21*). Thus, we set out to solve the structure for shutdown NM2A to determine how it forms the shutdown state and to aid interpretation of disease mutations.

## RESULTS

**Fig. 1.**
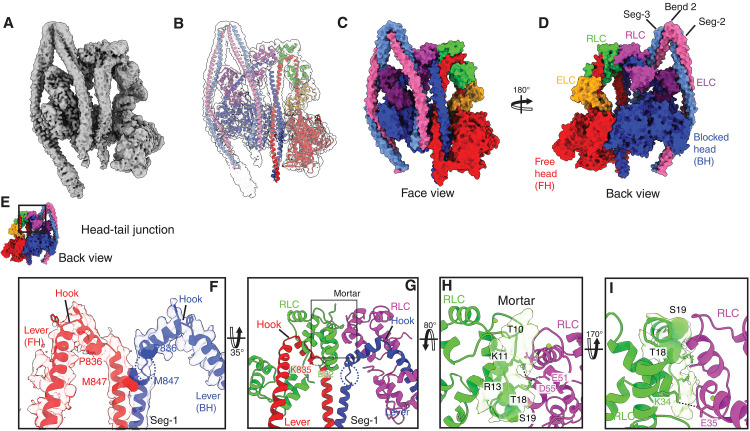
Structure of the interacting head region of shutdown NM2A and contributions of the free head RLC to the shutdown state. (**A**) Cryo–electron density map of the head region (face view; contour level = 0.02). (**B**) Structure of the head region fit into the density map. Surface representations of the front (**C**) and back (**D**) views of the structure. The HC is colored blue for the blocked head (BH), and red for the free head (FH; the ELC is colored dark purple for the BH, and yellow for the FH). The RLC is colored magenta for the BH, and green for the FH. The boxed region in (**E**), shown in (**F**), reveals how the levers from the FH and BH approach the head-tail junction via a kink in the α helix called the hook [bounded by two W (Trp) residues, shown as sticks]. The dashed blue circle indicates a region of unfolding of the α helix in the BH (movie S1B). (**G**) shows the head-tail junction, with both heavy and light chains included. Interacting residues between the RLC (FH) with the start of the coiled coil [Segment-1 (Seg-1)] are shown [Lys835 (K835):Glu32 (E32)]. No equivalent interactions are present for the RLC of the BH. The mortar is defined as residues 1 to 19 of the N-terminal FH RLC, which cements the two RLCs together. (**H**) shows the density and model for residues Ser19 (S19) to Thr10 (T10) in the mortar, revealing ionic interactions between Lys11 (K11) and Arg13 (R13) (FH RLC) with Asp55 (D55) and Glu51 (E51) (BH RLC), respectively. The phosphorylatable Ser19 and Thr18 residues are shown as spacefill. (**I**) shows an ionic interaction between the RLCs [Lys34 (K34): FH RLC with Glu35 (E35): BH RLC] just above the head tail junction.

Using cryo–electron microscopy (cryo-EM), we solved the structure of dephosphorylated human NM2A to a global resolution of 3.0 Å in the head region (Fig. 1, A to D, and fig. S1, A to G). The overall organization of the shutdown structure (Fig. 1, A to D) is similar to that previously reported for SMM structures obtained by cryo-EM (*12*–*14*). The structure of the motor domains reveals that both contain adenosine 5′-diphosphate.Pi (inorganic phosphate) (fig. S2, A to D) and that the backdoor, composed of residues Arg234 (R234) and Glu457 (E457), is shut, preventing Pi release (fig. S2, E and F). The overall organization of the nucleotide binding pocket is similar to that recently reported for sequestered autoinhibited interacting head structure of β-cardiac myosin (β-CM) (fig. S2G) (*22*, *23*) and is unlike that claimed for shutdown SMM (*12*).

### Head-tail junction: HCs

We first focus on the head-tail junction (Fig. 1, E and F), where the HCs from each lever emerge from the motor domains, bending at Trp825 to form the hook (WQWW:TypGlnTrpTrp) before ending at the invariant proline Pro836 (P836). The two HCs then associate to form the start of the coiled coil [Segment-1 (Seg-1)] at Met847 (Fig. 1, E and F, and movie S1).

The structure of the free head (FH) and blocked head (BH) HCs following Pro836 is different. In the FH, there is very little unwinding of the α helix (two residues to Gln839). This may be accounted for by the interaction between Glu32 in the FH RLC and Lys835 in the FH HC (Fig. 1G). In contrast, in the BH HC, unwinding is more extensive (four residues to Glu845) (dotted circle; Fig. 1F and movie S1B) following Pro836, and Glu32 in the BH RLC does not interact with Lys835 in the BH HC (Fig. 1G). The unwinding of the BH HC additionally accommodates the bent path of the BH lever, allowing the BH HC to enter the start of the coiled coil (Seg-1) in register with that of the FH. The two HCs are in register at the start of the coiled coil [Met847; (M847) Fig. 1F] and related by a 180° rotation around Seg-1 axis. Notably, the start of the coiled coil (Seg-1) takes a straight path down toward the FH as it emerges from the head-tail junction.

The overall structure of the head-tail junction in NM2A, while somewhat similar to that for full-length shutdown SMM (fig. S3, A to C), is markedly different to that reported for β-CM heavy meromyosin (HMM) (fig. S3D) (*22*, *23*). The BH levers take similar paths up to the hook for NM2A and β-CM; however, the structure of the hook is twisted backward and forms a more acute angle in β-CM compared to NM2A (fig. S3, E and F). In contrast, the FH levers take markedly different paths up to the hook (fig. S3, E and G). In β-CM, the FH lever is pulled closer toward the BH just after the converter, possibly as a result of the interaction between Lys611 (K611) in the BH motor domain and Asp143 (D143) in the FH ELC (*22*), absent in NM2A. Together with the altered hook structure of the β-CM FH and BH levers, the head-tail junction of β-CM is offset compared to NM2A, such that Seg-1 of the coiled coil takes a path across the BH for β-CM (fig. S3, E and G) and not straight down between the two heads as for NM2A, and as previously reported for SMM (*12*–*14*).

The lower resolution of the structure at the head-tail junction compared to other regions of the molecule (fig. S1, E and G) is likely due to the variable nature of the head-tail junction (fig. S3, H to J, and movie S2). As shown in the principal components analysis (PCA) morphs (fig. S3, H to J, and movie S2), the head-tail junction undergoes pronounced axial and lateral flexing as the entire molecule bends and twists. These coordinated motions highlight how this junction absorbs and accommodates the global contortions of the shutdown conformation. This is likely important in allowing distortion in the heads when both are bound to actin in the same thin filament (*24*) and in optimizing mechanical performance (*25*). In shutdown myosin, it is likely important for the head-tail junction to accommodate distortions resulting from the formation of the interacting heads motif by the motor domains, the paths of the two levers up to the head-tail junction, and the emergence of the coiled coil. Its variability mainly arises from the unfolding of the BH HC to allow for the twisting and translation of Seg-1, as it moves laterally to interact with Seg-3 and anteriorly/posteriorly to interact with the FH (fig. S3, H to J).

### Head-tail junction: RLC

The N-terminal residues of the FH RLC (residues 1 to 19) encapsulate the interface between the RLCs (Fig. 1, G and H, and movie S1C). We previously termed this region the mortar, as it appears to cement the two RLCs together (*13*). Adjacent to the head-tail junction, we traced the path of the backbone of the mortar to Thr10 (T10) (Fig. 1H), improving on previous structures for SMM (fig. S4, A to D). This revealed an ionic interaction between Arg13 (R13) (FH RLC) with Glu51 (E51) and Asp55 (D55) (BH RLC), explaining why the N-terminal region of the FH RLC takes a path upward between the two RLCs (Fig. 1H). Beyond Thr10, weaker unfilled density was consistent with the distal region adopting a range of positions. The two RLCs additionally interact outside of the mortar (Fig. 1I), similar to that found for SMM (fig. S4, A to D).

The phosphorylatable serine, Ser19, is exposed on the surface of the mortar. Its calculated solvent-accessible surface area is 227 Å^2^, which corresponds to a relative solvent accessibility of ~1.6, indicating that it is exposed on the protein surface (Fig. 1, H and L) (*26*). Its phosphorylation would push Ser19 away from and weaken the RLC:RLC interface because of electrostatic repulsion (fig. S4, E and F). Thr18, which can also be phosphorylated in cells resulting in diphosphorylated myosin (*27*, *28*), is similarly exposed and available for phosphorylation (Fig. 1H).

Using AlphaFold (*29*, *30*), we built a structure of the kinase domain of MLCK in complex with the RLC (fig. S4G). Docking of the AlphaFold model for the MLCK kinase domain into the mortar in the shutdown structure at the FH RLC site produced markedly fewer intermolecular steric clashes (634 clashes) than docking at the BH RLC site (2868 clashes; discussed below). This is consistent with improved steric accommodation at the FH RLC, showing that Ser19 is accessible to and can interact with the kinase domain of MLCK with minimal steric clashes (fig. S4, H and I, and movie S1D). Phosphorylation of Ser19 in the mortar would increase the mobility and variability of the head-tail junction, which is likely to destabilize the shutdown state.

### Interactions of Segs-1 and -3 with the head region and each other

**Fig. 2.**
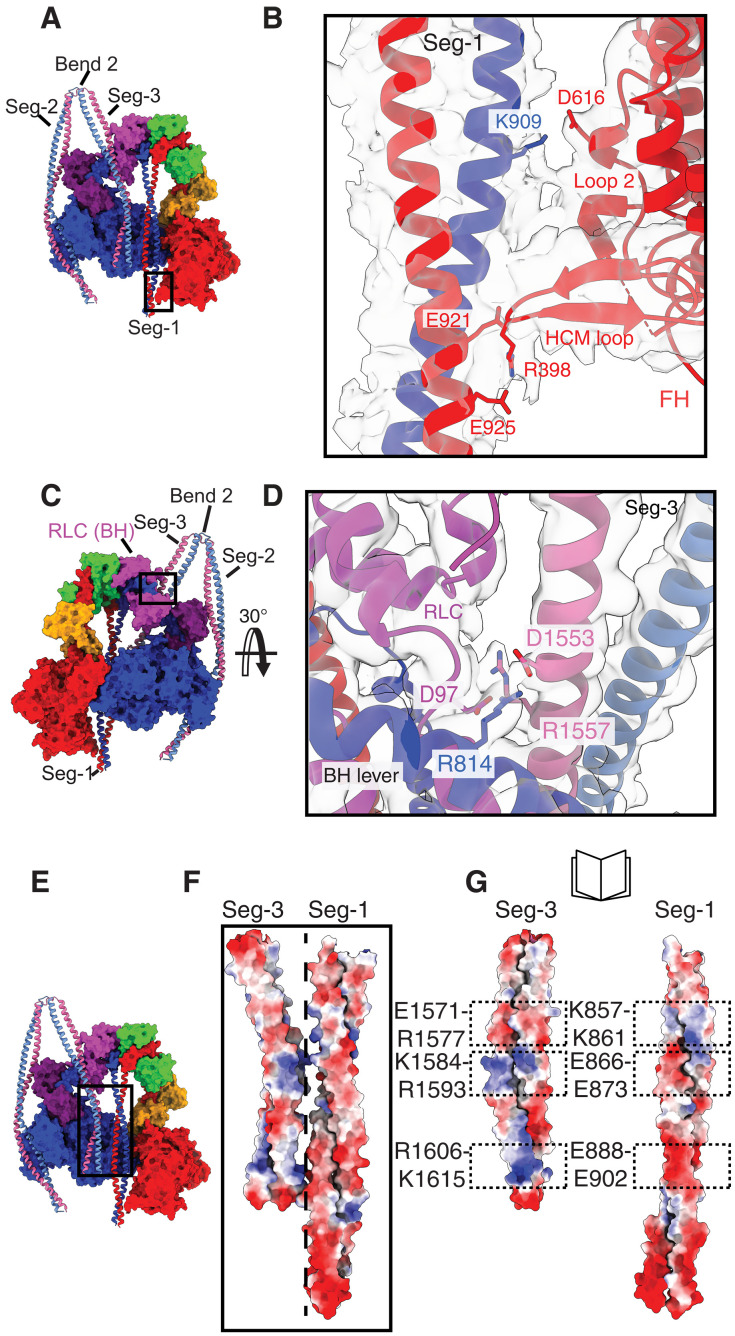
Interactions of Segs-1 and -2 with the motor domain and Segs-1 and -3 with each other. (**A**) Colored surface structure (face view) with the coiled coil shown as ribbons. The boxed region is expanded in (**B**) to show the ionic interactions between Lys909 (K909)(Seg-1) and Asp616 (D616) (loop 2) and Glu921 (E921)and Glu925 (E925) (Seg-1) with Arg398 (R398) (HCM loop). Zoned map at contour level 0.02. (**C**) Colored surface structure (back view) with coiled coil shown as ribbons. The boxed region is expanded in (**D**) to show interactions between Seg-3 and both the RLC and lever of the BH [Asp1553 (D1553); Arg814 (R814), Asp97 (D97) (BH RLC):Arg1557 (R1557)]. Zoned map at contour level 0.02. (**E**) Colored surface structure (front view), with coiled coils shown as ribbons to show the positions of Seg-1 and Seg-3 as they pass across the BH (boxed region). Expanding the boxed region in (E) in (**F**) shows the multiple potential ionic interactions between Seg-3 (BH) and Seg-1 (FH), each colored by electrostatic potential. (**G**) The interface between Seg-3 and Seg-1 is opened up (as a book) to display the electrostatic seam between the two pairs of coiled coils. Dashed boxes show patches of opposite charge between Segs-1 and -3.

After Seg-1 emerges straight down from the head-tail junction, it runs across the BH close to the BH-FH interface, continuing downward to interact with the FH loop 2 and HCM (hypertrophic cardiomyopathy) loop (Fig. 2, A and B). In contrast, Seg-1 in β-CM does not emerge straight down from the head-tail junction but at an angle such that it runs across the middle of the BH and interacts with its mesa trail (fig. S5, A to C) (*31*).

In addition, in this region of NM2A, Seg-3 emerges from bend 2 to pass down besides Seg-1 (Fig. 2A). Its initial path is likely influenced by interactions between charged residues in Seg-3 and in the lever and BH RLC (Fig. 2, C and D). Seg-3 then begins to strongly interact with Seg-1 through a series of multiple ionic interactions (Fig. 2, E to G) such that there is a tight association between these two regions of coiled coil.

While the path of Seg-3 in NM2A appears similar to that of Seg-1 in β-CM, there are key differences. Seg-3 of NM2A only makes two initial interactions with the BH (fig. S5, A and C), compared to multiple interactions made between Seg-1 in β-CM (HMM shutdown structure) and the mesa trail of the BH (fig. S5, B and E). For the first of these in NM2A [Arg442 (R442) (loop O):Glu1593 (E1593) (Seg-3)], equivalent interactions are also found in SMM (fig. S5D) and β-CM [Lys450 (K450) (loop O):Glu875 (E875) (Seg-1)]. For the second [Lys656 (K656) (helix W):Asp1600 (D1600) (Seg-3)], an equivalent interaction is only found in β-CM [Thr660 (T660) (helix W):Gln892 (Q892) (Seg-1)] and not SMM. In this region, Seg-1 and Seg-3 in NM2A have already begun to interact, Seg-3 does not lie close to the BH, and no further interactions with the BH occur. In contrast, Seg-1 in β-CM continues to run close across the BH, and residues in Ring-1 (fig. S6: residues 894 to 905) and immediately downstream make multiple additional interactions with residues in the mesa of the motor domain. Similar interactions are also found in shutdown molecules in the cardiac myosin filament (*32*).

It is possible that Seg-1 in NM2A and SMM could interact with the BH in a similar way to β-CM, if Seg-3 was absent and if a change to the overall conformation of the head-tail junction in NM2A enabled Seg-1 to take the angled path observed for β-CM. The potential for this is evidenced by relatively high conservation of the HC sequence for residues in Ring-1 (residues 892 to 903 in NM2A) and immediately downstream (to residue 914) between the three myosin isoforms (fig. S6) and residues in the mesa (between residues 513 to 550 in NM2A; fig. S6). However, in the full-length NM2A, the small number of contacts between Seg-3 and the BH (fig. S5, C and D), together with the tight association between Segs-1 and -3 in shutdown NM2A, suggest that the interactions between the coiled coils in these two segments predominantly dictate their paths in this region of the molecule. Overall, the straight path of Seg-1 and its interactions with loop 2 of both BH and FH, together with the strong interactions between Segs-1 and -3, and the interactions of Seg-3 with the BH RLC and lever help to explain the path of Segs-1 and-3 in the head region.

### Seg-2 and its interactions with the BH, bend 2, and the N-terminal BH RLC (latch)

Below the myosin heads, Seg-1 continues down to bend 1. Bend 1 was identified as a region of unwinding around residue Ala1156 (A1156), using a combination of AlphaFold modeling of the missing coiled segments (the density in this region is too weak to fit the structure) and cross-linking mass spectrometry data (fig. S7 and see Materials and Methods).

**Fig. 3.**
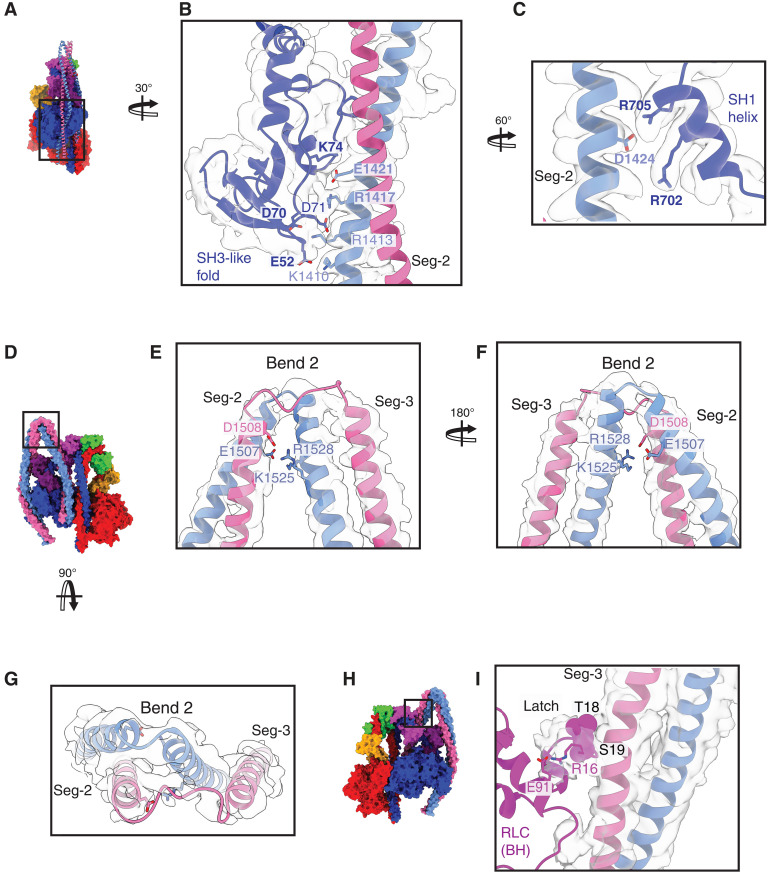
Interactions between Seg-2 and the BH motor domain, formation of bend 2, and the N-terminal region of the BH RLC (latch). (**A**) shows a side view of the motor domain of the BH (colored surface structure), revealing how Seg-2 of the coiled coil sits in a groove within the BH motor. (**B**) One view of the boxed region in (A) shows the ionic interactions between residues in the N-terminal SH3-like fold [Glu52 (E52), Asp70 (D70), Asp71 (D71), and Lys74 (K74)] with Seg-2 [Lys1410 (K1410), Arg1413 (R1413), Arg1417 (R1417), and Glu1421 (E1421)] (zoned map at contour level 0.02). (**C**) A second view shows the ionic interactions of specific residues [Arg701 (R701) and Arg704 (R704)] in the SH1 helix of the motor domain (BH) with Seg-2 [Asp1424 (D1424)] (zoned map at contour level 0.1). Residues shown in bold in (B) and (C) are mutated in MYH9-related disease. (**D**) Face view of bend 2 (colored surface structure). An expanded view of the boxed region is shown in face view (**E**), back view (**F**), and top view (**G**) to show the local unwinding of the coiled coil and the ionic interactions that stabilize this bend (zoned map at contour level 0.1). (**H**) Back view (colored surface structure). (**I**) shows the boxed region in (H) to show residues Ser19 (S19) to Arg16 (R16) in the N-terminal latch (defined as residues 1 to 19 of the BH RLC) and their proximity to Seg-3. The phosphorylatable Ser19 and Thr18 residues are shown as spheres. (Zoned map at contour level 0.02).

The coiled coil then returns back toward the BH as Seg-2. It first makes key interactions with residues from the N-terminal Src homology 3 (SH3)–like fold and the SH1 helix of the BH motor domain, sitting within a small groove formed by these two regions (Fig. 3, A to C), similar to our findings for SMM (*13*). The improved resolution of our NM2A structure allows us to identify key interacting residues between the motor and Seg-2 in this region confidently. Missense mutations in any one of the 11 interacting residues (8 residues in the motor Glu52, Asp70, Lys74, Arg705, and Arg702; E52, D70, K74, R805 and R702 respectively) and 3 in the tail [Glu1421, Arg1417, and Asp1424 (E1421, R1417 and D1424 respectively)] (Fig. 3, A and B) have been linked to MYH9 disease, highlighting the importance of this region of the structure in stabilizing the shutdown state (*15*, *33*). In agreement, we previously reported that missense mutations in NM2A in one of these key residues [Asp1424 (D1424)], commonly mutated in MYH9 disease (*33*), destabilized the shutdown state (*15*). In our NM2A structure, we additionally resolved the complete N-terminal region upstream of the SH3-like fold for both the BH and FH (fig. S8), revealing how this region of the structure lies across the motor domain. MYH9-disease–related mutations in this region likely affect the subsequent positioning of the SH3-like fold, destabilizing its interaction with Seg-2.

The interactions we observed between Seg-2 and the BH are similar to those we previously suggested would be present in SMM from our pseudoatomic model at lower resolution (fig. S9, A to C and E to G) (*13*). However, a more recent SMM structure [7MF3; (*12*)] does not show similar interactions (fig. S9, D and H). To try to understand this, we compared our coiled-coil structure in NM2A to that in SMM (7MF3) and noticed that the coiled coil in 7MF3 goes out of register in Seg-2 (fig. S10, A and B) (*12*). A transverse view through the coiled coil in a region of Seg-2 from NM2A (residues 1408 to 1411) shows how the two chains are in register, with sufficient density to fit the side chains for the *a* [Tyr1408 (Y1408)] and *d* [Leu1411 (L1411)] residues, demonstrating that they are embedded within the hydrophobic seam (fig. S10C). This is not the case in the equivalent region of 7MF3; both *a* [Tyr1421 (Y1421)] and *d* [Leu1424 (L1424)] residues are not in register, and neither are embedded within the hydrophobic seam (fig. S10D). Thus, it is likely that the coiled coil in 7MF3 was not built correctly and cannot therefore be used to identify interactions between Seg-2 and Seg-3 and the rest of the molecule.

As Seg-2 continues toward bend 2, it first makes two ionic interactions with the ELC (fig. S11), similar to our previous findings for SMM (*13*). These interactions may bias the path of Seg-2 toward bend 2. The two residues in Seg-2 (E1472 and R1482) that interact with the ELC are not conserved in Seg-2 of β-CM (fig. S11, C and D). This could explain why, in isolated molecules of β-CM, the coiled coil may weakly interact with the motor domain in the region of the groove (*34*) but then does not make any further interactions with the motor and bend 2 fails to form.

The resolution of our NM2A structure reveals that bend 2 in NM2A is facilitated by a small unwinding of the coiled coil rather than a bend at a single residue (Fig. 3, D to G). The unwinding we observe in NM2A is similar to that for SMM, although we attributed bend 2 to a single glycine residue (*13*). In NM2A, 10 residues in the FH α helix are unwound at bend 2 [Met1510 (M1510) to Ser1519 (S1519)], and G1516 (Gly1516, equivalent to the single glycine residue identified as bend 2 in SMM) is within this region. However, only two residues in the BH α helix [Lys1513 (K1513) to Asp 1514 (D1514)] are unwound. Ionic interactions between Seg-2 and Seg-3 help to stabilize bend 2 (Fig. 3, E and F).

As Seg-3 passes back down to interact with Seg-1 after bend 2, it is likely to interact with the latch [defined as residues 1 to 19 of the BH RLC (*13*)]. We resolved the path of the backbone of this region of the RLC up to Arg15 (R16), an improvement on our previous SMM structure (fig. S12, A to D) (*12*–*14*). The unfilled density beyond this was weaker, suggesting that this distal region can adopt various conformations, all close to Seg-3. Phosphorylation of Ser19 in the latch would cause side-chain clashes and repulsion by negatively charged residues in Seg-3 (fig. S12, E to G) releasing the latch from its shutdown position. Docking the kinase domain from MLCK showed that its access to Ser19 is sterically blocked by Seg-3 (fig. S12, H and I) in contrast to our finding for the mortar (see discussion above). This suggests that S19 in the mortar (FH RLC) is phosphorylated first, and subsequent disruption of the shutdown state then allows S19 in the latch (BH RLC) to be phosphorylated, as has been previously speculated for SMM (*13*, *14*). This contrasts with other reports that suggests that Ser19 in the mortar is less accessible but which did not explore the ability of MLCK to bind to the latch and mortar (*12*, *35*).

### Full-length shutdown NM2A

**Fig. 4.**
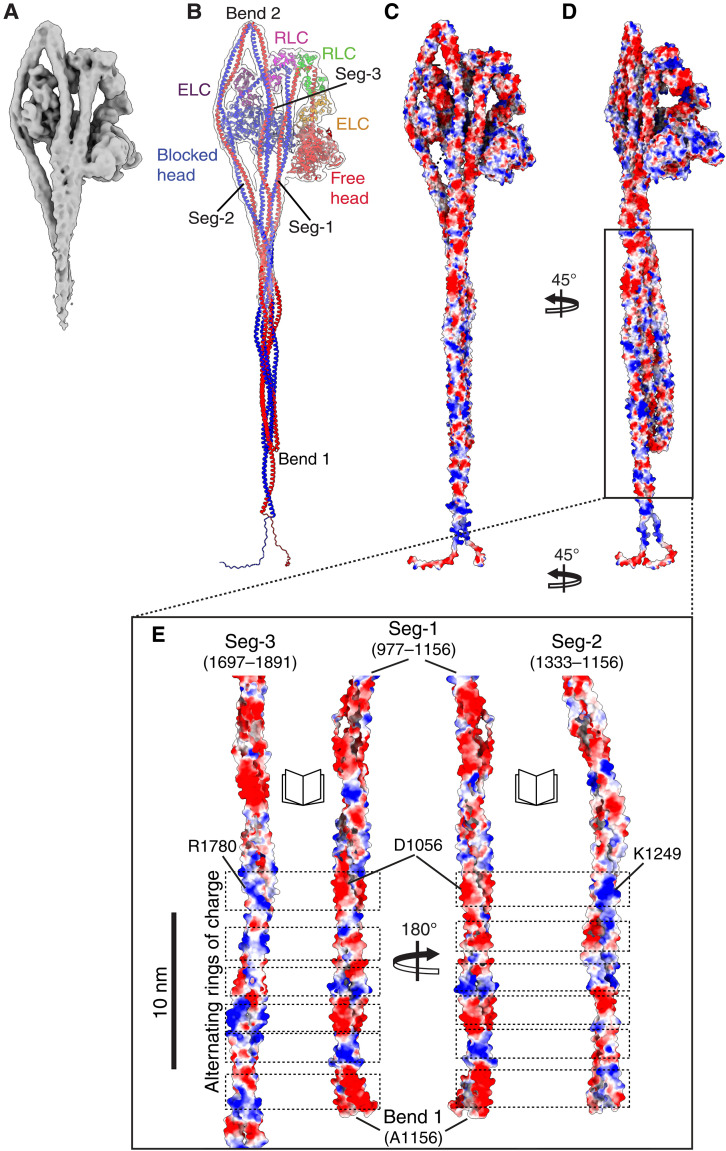
Structure of the whole 10*S* complex of NM2A and the flat ribbon arrangement of its coiled-coil tails. (**A**) Cryo–electron density map of the full-length molecule (front view). (**B**) The structure of whole molecule fit into the density map. The structure has been colored by chain in the same way throughout: Red indicates the HC for the motor, lever, and the first part of the coiled coil (Seg-1) for the FH; and blue for the BH [chain assignment as (*13*) for SMM]. ELC: dark purple for BH and orange for FH. RLC: green for FH and magenta for BH. (**C**) Surface representation (face view) and (**D**) side view of the structure in (B) colored by electrostatic potential. (**E**) shows the interfaces between Seg-3, Seg-1, and Seg-2, opened up (as a book) to display the electrostatic seam between the two pairs of coiled coils (Seg-1:Seg-3 and Seg-1:Seg-2). Dashed boxes show patches of opposite charge between Segs-1 and -3 and between Segs-1 and -2.[R1780 (Arg1780), D1056 (Asp1056), K1249 (Lys1249), A1156 (Ala1156)].

We additionally solved the structure of an extended region of shutdown NM2A to a global resolution of 6.3 Å (Fig. 4, A to D, and fig. S1). This extends the structure to about two thirds of the way along the coiled-coil tails. The high mobility of the distal region (movie S3) prevented us from solving the entire structure from the density map. However, we used AlphaFold modeling to build in the missing coiled-coil segments outside the density map and used cross-linking mass spectrometry to validate this model (see Materials and Methods). The structure of the full-length molecule shows how the three segments of the coiled coil come together and interact in NM2A (Fig. 4, C to E), as we suggested for SMM, to form an untwisted ribbon structure (*13*). Seg-1 lies between Seg-2 and Seg-3. A coulombic plot shows the alternating positively and negatively charged regions that enable the interaction between Segs-1 and 3 in this region (Fig. 4E).

## DISCUSSION

### Model for activation of NM2A by RLC phosphorylation

**Fig. 5.**
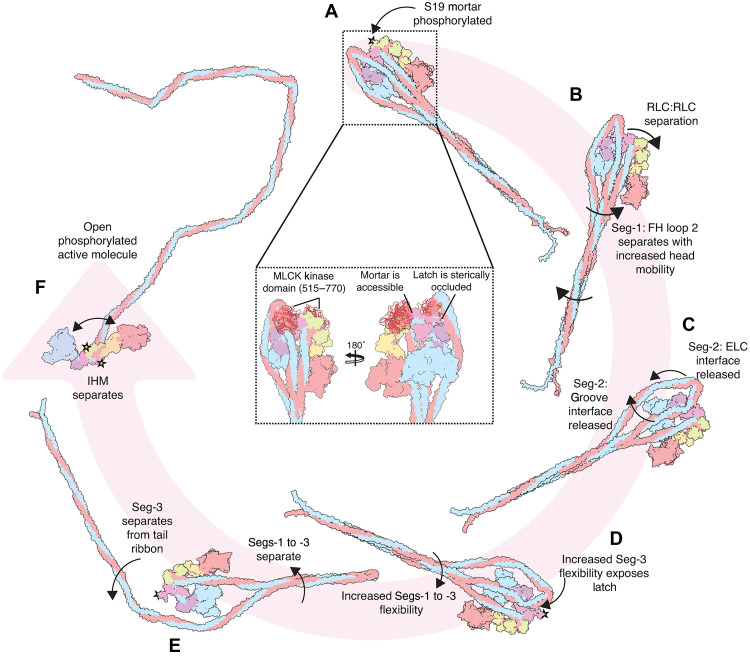
Possible sequence of 10*S* to 6*S* transition. (**A**) Intact 10*S* molecule is phosphorylated at the mortar (phosphorylation site indicated by the gold star). Insets show the different accessibility of the MLCK kinase domain to the RLC N-terminal extensions of FH and BH. (**B**) Destabilization of the RLC-RLC interface increases the mobility of Seg-1 at the head-tail junction, allowing Seg-1 to separate from the FH. (**C**) Increased mobility of the tail segments allows Seg-2 to be released from the SH3 groove and ELC interface. (**D**) The increased mobility of all three segments of the tail exposes the latch to become accessible to MLCK and then phosphorylated (gold star indicates phosphorylation site). (**E**) With the latch phosphorylated, Seg-3 is released from Seg-1, and then the molecule is completely unfolded and active (**F**).

If Ser19 in the mortar is phosphorylated first, as our data seem to indicate, then we can build a picture of how the shutdown molecule would become destabilized when the RLCs are phosphorylated (Fig. 5). Phosphorylation of FH RLC Ser19 (Fig. 5A) would destabilize the mortar, which, in turn, would destabilize RLC-RLC interactions at the head-tail junction (Fig. 5B). This would lead to an increased mobility of the head-tail junction, and thus Segs-1 and -3, which are closely associated (Fig. 5C). This, in turn, would increase mobility of Seg-2, pulling Seg-2 out of the N-terminal groove formed by the SH3-like fold and the SH1 helix and away from the ELC, leading to the release of Seg-2 from the IHM (interacting head motif). The increased mobility of all segments of the tail would expose the latch, allowing access of MLCK to Ser19, and its phosphorylation (Fig. 5D). With the latch phosphorylated, Seg-3 would be released from the tail ribbon (Fig. 5E), leading to separation of all three tail segments and dissolution of the IHM resulting in an active open molecule (Fig. 5F).

The shutdown NM2A structures presented here reveal how NM2A is shutdown in its dephosphorylated state, the key residues that stabilize this state, and how phosphorylation activates the molecule. The roles of key residues in stabilizing this shutdown state help us understand how mutations could destabilize it, leading to disease.

## MATERIALS AND METHODS

### Protein expression and purification

NM2A protein was obtained by coexpression of FLAG-tagged human NM2A HC (MYH9 coding sequence: National Center for Biotechnology Information: NG_011884.2) with bovine nonmuscle myosin RLC (myosin light chain 9 (MYL9): 95% identical in amino acid sequence to human) and chicken nonmuscle myosin ELC (myosin light chain 6 (MYL6): 90% identical in amino acid sequence to human) as reported previously (*15*), using Sf9 cells. The protein was purified using M2 FLAG affinity gel (Sigma-Aldrich) as described previously (*15*, *36*). NM2A expressed in this way is not phosphorylated (*37*).

To form the shutdown state, Mg.ATP was added to the purified NM2A, before dilution into a low–ionic strength solution. Final conditions were 140 mM KCl, 10 mM Mops (pH 7.2), 0.1 mM EGTA, 2 mM MgCl_2_, and 1 mM ATP. The 10*S* particles (at a concentration of 1 μM) were cross-linked using 1 mM bis(sulfosuccinimidyl)suberate (Thermo Fisher Scientific) for 30 min at 25°C and then quenched with the addition of pH 8.0 tris buffer to a final concentration of 100 mM to stabilize the shutdown state.

### Grid preparation and cryo-EM data acquisition

Grids were prepared with a Vitrobot Mark IV (Thermo Fisher Scientific), applying 3 μl of NM2A (0.6 μM) to UltrAuFoil R1.2/1.3 Gold 300 mesh grids (Agar Scientific, UK) glow discharged for 90 s before use (PELCO easiGlow discharge unit, Ted Pella Inc.). Grids were blotted with Whatman no. 42 Ashless filter paper (Agar Scientific, UK) for 3 s at force 6, at a temperature of 8°C and 100% humidity, and flash frozen in liquid ethane. Data were recorded on a FEI Titan Krios (Astbury Biostructure Laboratory, University of Leeds) operating at 300 kV equipped with a TFS Falcon 4i direct electron detector and a BioQuantum energy filter (Gatan, CA, USA). Micrographs were recorded with the EPU automated acquisition software at ×96,000 nominal magnification, giving a final object sampling of 0.82 Å per pixel, a total dose of 37 electrons/Å^2^, and a target defocus range between −0.9 and −3.0 μm (*38*). A total of 27,549 micrographs was acquired in two microscope sessions using the same collection parameters (table S1).

### Cryo-EM image processing

Image processing was carried out using CryoSPARC v4.6.0 (*39*). Drift-corrected averages of each movie were created using the Patch-Based Motion Correction in CryoSPARC, and the defocus of each was determined using Patch CTF (*40*). Micrographs were curated to exclude micrographs with excessive ice thickness, poor contrast transfer function (CTF) fit, and large full-frame motion. Particle picking was performed using a combination of Blob Picker functionality and the Topaz wrapper in CryoSPARC (*41*, *42*). Particles were extracted in a box size of 480 by 480 pixels, centered on the interacting heads motif (IHM) region, and were classified using two-dimensional (2D) classification in CryoSPARC. Classes that contained features reflective of shutdown myosin structure were selected and taken forward for further classification in CryoSPARC. Four initial 3D volumes were produced using ab initio reconstruction in CryoSPARC. This model was refined with several rounds of heterogeneous and nonuniform refinement. The final particle stack contained 349,374 particles from 20,755 micrographs. Reference-Based Motion Correction and a final 3D refinement yielded a map for the head region of the full-length molecules that had a 3.0-Å global resolution determined using the gold standard Fourier shell correlation (FSC) reported to FSC = 0.143 (fig. S1). The EM density map was sharpened using a negative B-factor that was automatically determined in CryoSPARC using a Guinier plot. Local resolution was estimated using CryoSPARC.

3D variability analysis was performed in CryoSPARC to assess structural heterogeneity in the shutdown NM2A molecules. The refined particle stack was used as input for the 3D variability analysis, which computed the first three principal components describing the major motions present in the data. Volume series representing the positive and negative extremes of each component were generated in CryoSPARC and visualized in ChimeraX to produce the PCA movies.

To obtain a structure of the full-length NM2A molecule, particles used to generate the 3.0-Å global resolution map of the NM2A head region in CryoSPARC were reextracted in a box size of 1024 by 1024 (binned twofold into a 512 by 512 box). An initial 2D classification was performed. Good particles were first classified in 2D, followed by ab initio reconstruction and nonuniform refinement in CryoSPARC. The final global resolution of the NM2A shutdown whole-molecule map was 6.3 Å (from 15,167 particles), determined using the gold standard FSC reported to FSC = 0.143 using CryoSPARC. Figures and videos were generated in ChimeraX (*43*) or Chimera (*44*).

### Model building and refinement

To interpret the 3.0-Å map for the IHM region, we created an atomic model for the myosin heads using ModelAngelo (*45*) to automatically build into the map, using the sequences of the HC, RLC, and ELC used in our expression construct. Mg^2+^ was present in both the nucleotide binding pocket and in the RLC. Unbuilt portions were performed manually using the SMM structure (*13*) as a homology model and then using flexible fitting using ISOLDE (*46*). Coot (*47*) was used to address bond length and geometry issues introduced by molecular dynamics simulation of model building using ISOLDE.

To generate a full-length NM2A model, a full-length SMM homology model based on the whole SMM structure was used in ISOLDE to build into the extended map. Chains C, D, E, and F were built to the same extent as in the head-only model. Segs-1, -2, and -3 were built into density from residues 847 to 1072, 1235 to 1516, and 1517 to 1794, respectively. AlphaFold3 was used to generate three model coiled-coil segments for the missing tail sequences, which each contained 14 residues that overlapped the tail sequences of Segs-1, -2, and -3 of our pseudoatomic model of the IHM region. These models were superposed onto the existing coiled-coil tail segments, and then the “fit selection only” flag was used in rigid body fitting as described for our 9-Å map of the whole SmM shutdown molecule (*13*). This allowed the introduction of the bends into the coiled-coil tail and for them to lie in plane as seen in the density map. With Segs-1 and -2 lying antiparallel to one another, the point at which their sequences coincided was found to occur at A1169, so bend 1 was created at this point. The chains were joined, and duplicated sequence was deleted, followed by manual refinement in Coot. The junction made between Segs-1 and -2 at bend 1 implies that in Segs-2 and -3, chain G is a continuation of chain A, and chain H is a continuation of chain B.

Cross-linking mass spectrometry distance restraints (see below) together with ISOLDE were used to evaluate the compatibility of the final full-length model with experimentally observed proximities. Identified BS3 cross-links were mapped onto the final model and evaluated using a maximum Cα-Cα distance cutoff of 27 Å, consistent with the spacer arm length of BS3 (~11.4 Å) plus the lengths of lysine side chains and conformational flexibility (*48*). Cross-links were mapped onto the model and assessed against expected linker length constraints, providing validation of the proposed architecture and supporting the placement of structural features; notably, the distribution of satisfied and violated cross-links supports the presence and location of bend 1, consistent with the observed structural arrangement. As the entirety of each HC can be built, the full-length model follows the same chain nomenclature as above but with chains G and H being built as a continuation of chains A and B, respectively.

The structure of the bovine RLC (MYL9, residues 1 to 173) in complex with the human MLCK (kinase domain 491 to 746), which we term the RLC_MLCK complex, was generated using the AlphaFold3 server (https://alphafoldserver.com/) (*30*). The bovine RLC sequence was used, for consistency with the NM2A structure obtained here. The top scoring model was aligned using residues 17 to 23 of the RLC N-terminal extension in the RLC_MLCK complex onto each RLC chain (chains E and F; residues 17 to 23) of our 10*S* structure using the “Matchmaker” command within USCF ChimeraX. With the RLC_MLCK complex docked into place, the RLC chain from the RLC_MLCK complex was deleted to yield the MLCK kinase domain in complex with both the FH and BH RLC chains of our 10*S* structure.

### Cross-linking mass spectrometry

Cross-linking mass spectrometry was performed as previously described (*48*). Briefly, cross-linked samples were reduced (20 mM dithiothreitol), alkylated (40 mM indole-3-acetic acid), acidified, and digested on S-Trap columns (ProtiFi) with trypsin at 47°C for 90 min. Peptides were desalted, concentrated, and reconstituted in 0.1% formic acid. Liquid chromatography–mass spectrometry was carried out on an Orbitrap Eclipse Tribrid with a Vanquish Neo ultrahigh-performance liquid chromatography, using a PepMap C18 trap and EASY-Spray C18 analytical column with a 95-min, 2 to 50% acetonitrile gradient. Data-dependent acquisition was used (MS1: 120,000 resolution, 380 to 1450 mass/charge ratio; MS2: HCD (higher-energy collisional dissociation), 60,000 resolution, dynamic exclusion of 30 s). Data were analyzed in Proteome Discoverer v3.0 (XlinkX) and Merox v2.0.1.4 with a custom FASTA [precursor: 5 parts per million (ppm), fragment: 10 ppm, trypsin with two miscleavages, carbamidomethyl Cys fixed, Met oxidation and N/Q deamidation variable, false discovery rate <1%, and score >50].
