# Supplementary material for: Cryo-EM structure of shutdown human nonmuscle myosin 2A
Source: Sci Adv. 2026 Apr 17;12(16):eaed1858. doi: 10.1126/sciadv.aed1858 (PMC13089345; doi:10.1126/sciadv.aed1858)
Supplement: Supplementary file 1 — Figs. S1 to S12 Table S1 Legends for movies S1 to S3 [file sciadv.aed1858_sm.pdf]

Science Advances
AAAS

Supplementary Materials for

Cryo-EM structure of shutdown human nonmuscle myosin 2A

David Casas-Mao *et al.*

Corresponding author: Michelle Peckham, m.peckham@leeds.ac.uk

Sci. Adv. **12**, eaed1858 (2026)
DOI: 10.1126/sciadv.aed1858

The PDF file includes:

Figs. S1 to S12
Table S1
Legends for movies S1 to S3

Other Supplementary Material for this manuscript includes the following:

Movies S1 to S3

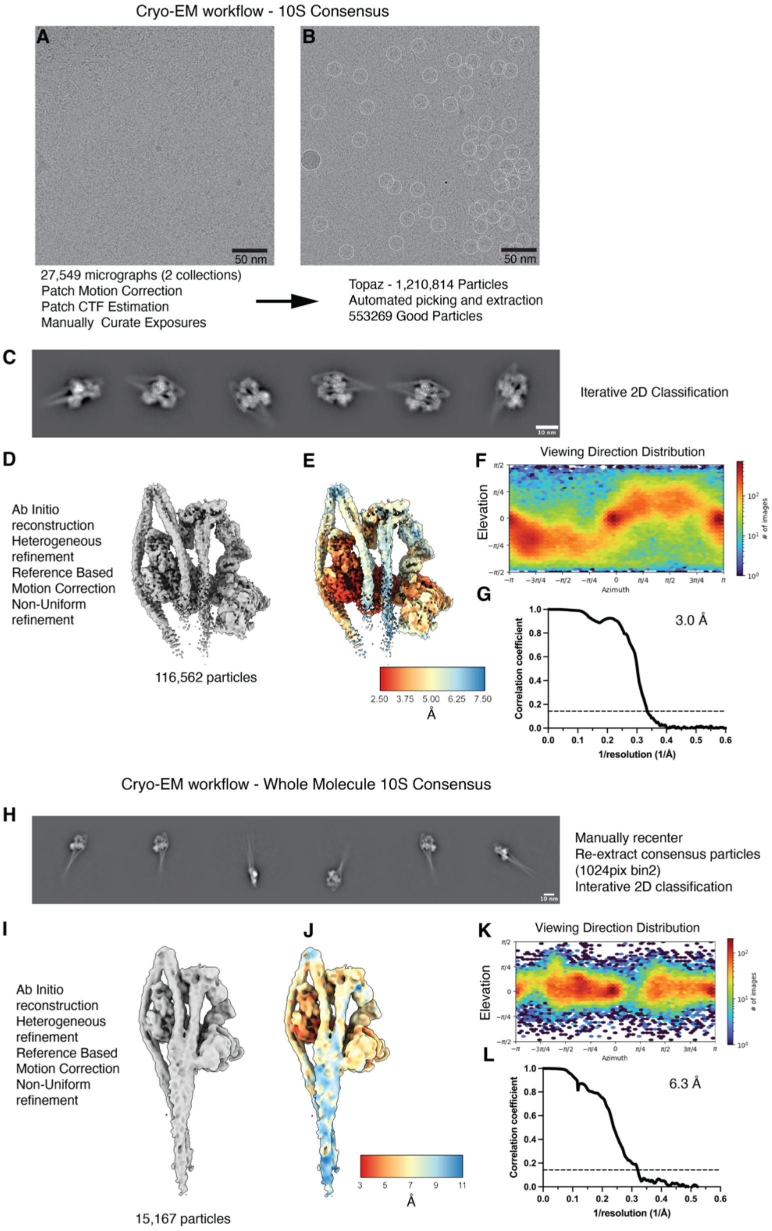

Fig. S1. NM2A Processing Workflow.

(A) Representative micrograph of shutdown NM2A molecules. **(B)** Representative micrograph with particle picks. **(C)** Representative 2D classes (Scale bar 10nm). **(D)** Final NM2A shutdown heads region cryo-EM map (deposited as EMD-55354). **(E)** Local resolution of map shown in D. **(F)** Angular distribution of particles in the heads region 3D reconstruction. **(G)** FSC curve of the heads region reconstruction, illustrating 3.0 Å resolution at 0.143 FSC. **(H)** Representative 2D classes of the whole molecule. Particles were extracted in a 1024 pixel box and binned twofold. (Scale bar 10nm) **(I)** Final NM2A shutdown whole molecule cryo-EM map (deposited as EMD-55382). **(J)** Local resolution of map shown in I. **(K)** Angular distribution of particles contributing to the whole molecule 3D reconstruction. **(L)** FSC curve of the whole molecule reconstruction, illustrating 6.3 Å resolution at 0.143 FSC.

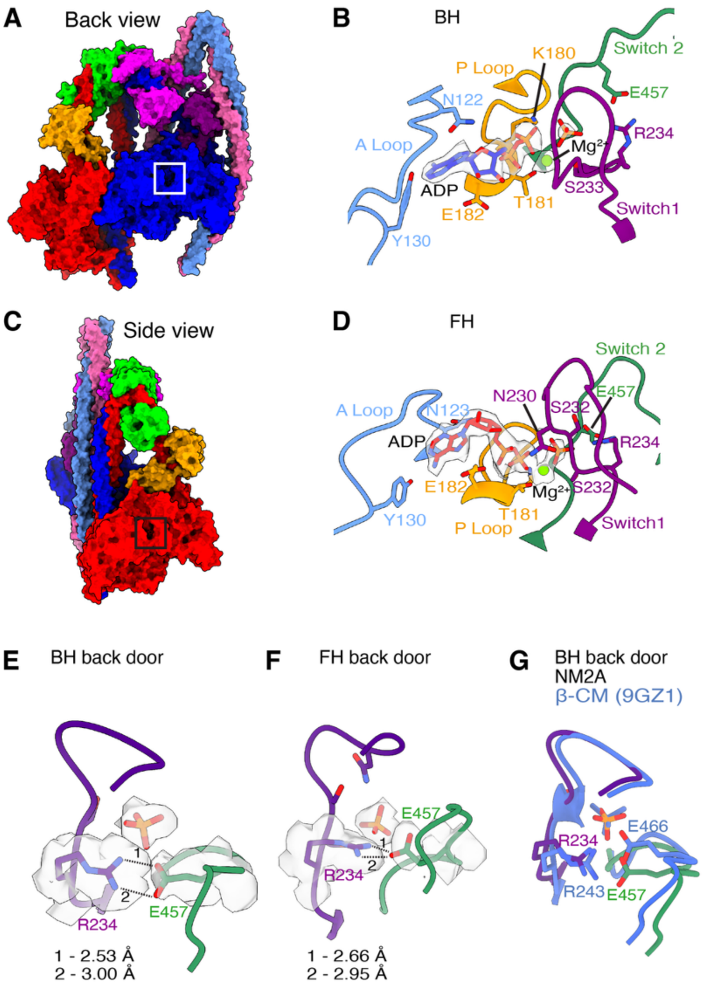

Fig. S2. The nucleotide binding pocket in shutdown NM2A

(A) Colored surface view of the heads region (back view) to show the location of the blocked head (BH) nucleotide pocket. **(B)** Zoomed view of region boxed in A. Side chains of interacting residues in the A Loop, P Loop, Switch 1 and Switch 2 are indicated. ADP.Pi and Mg^{2+} are fit to segmented density. **(C)** Colored surface view of the heads region (side view) to show the location of the Free Head (FH) nucleotide pocket. **(D)** Zoomed view of region boxed in C, with equivalent interacting loops and residues indicated as in B. **(E-G)** Salt bridge blocking Pi release and corresponding bond lengths in the BH and FH back door. **(G)** Alignment of NM2A and β-CM (PDB:9GZ1) for the BH back door.

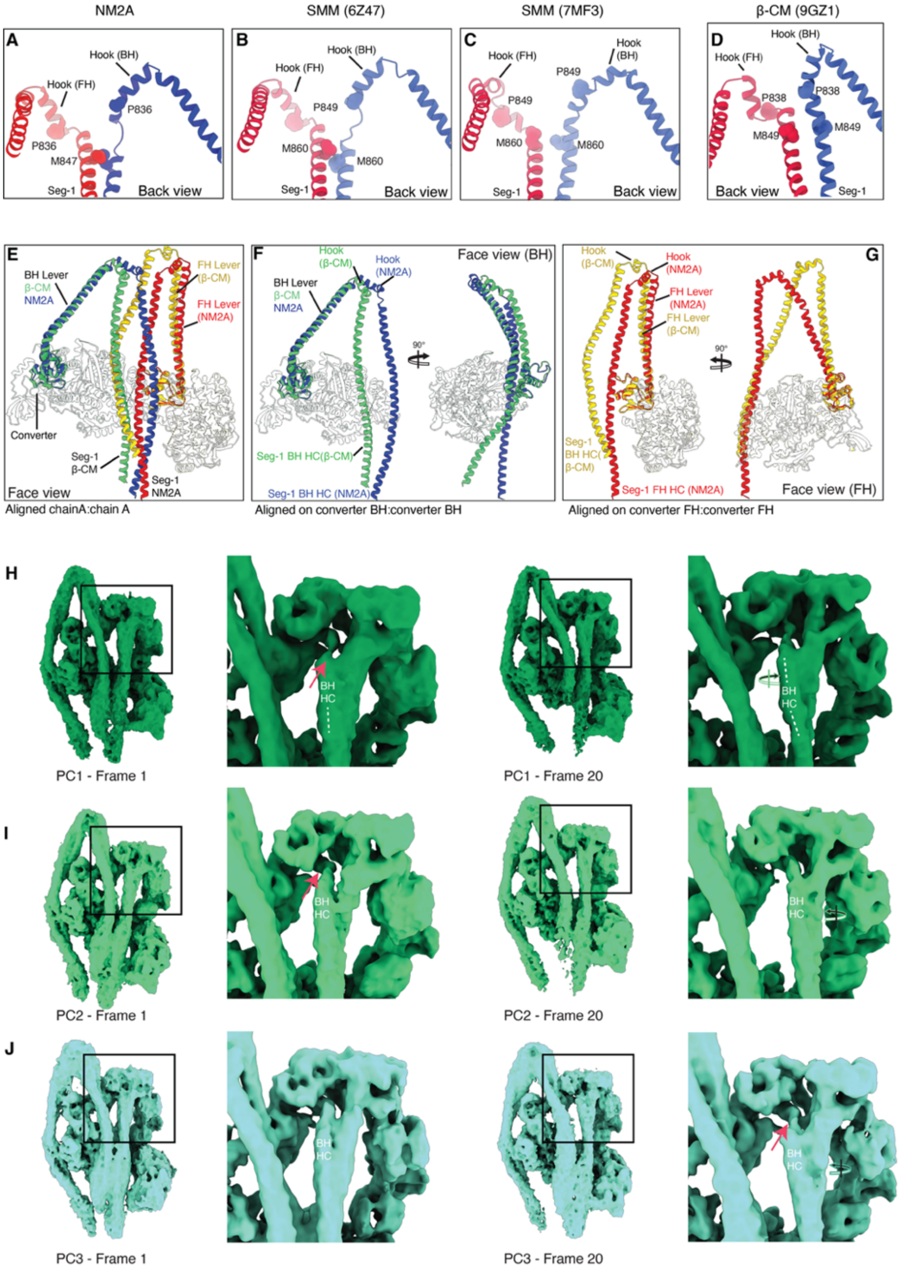

Fig S3: Comparison of β-CM and NM2A levers and Head-Tail junction. (**A**) Back view of the Head-Tail junction of NM2A. Light chains are hidden and the side chains of the invariant proline that marks the start of the Head-Tail junction together with the conserved methionine (M860) are shown as spheres. (**B-D**) Same representation as in A for 6Z47 *(13)*, 7MF3 *(12)*, and 9GZ1 *(22)* respectively. A comparison with the additional SMM structure (6XE9 (1*4*)) was not performed here but it shows similarity to 6Z47. (**E**) Alignment of shutdown β-CM HMM and shutdown NM2A whole molecule HCs in the region of the heads. Light chains are hidden and heavy chain (HC) residues before the converter are grayed out for clarity. (**F**) Alignment of β-CM and NM2A BH HCs. Alignment was performed on the converter to emphasize the divergent path of the hook and Seg-1. (**G**) Alignment of β-CM and NM2A FH HCs. Alignment was performed on the converter to emphasize the divergent path of the LCD, hook, and S1. **H, I** and **J** show the first and last frame of the 3 component Principal Component Analysis of the 3D Variability Analysis performed in CryoSparc (see methods). Arrows indicate the twisting and lateral movement of Seg-1 as it emerges from the head-tail junction across the population of conformations. (See Supplemental movie 2).

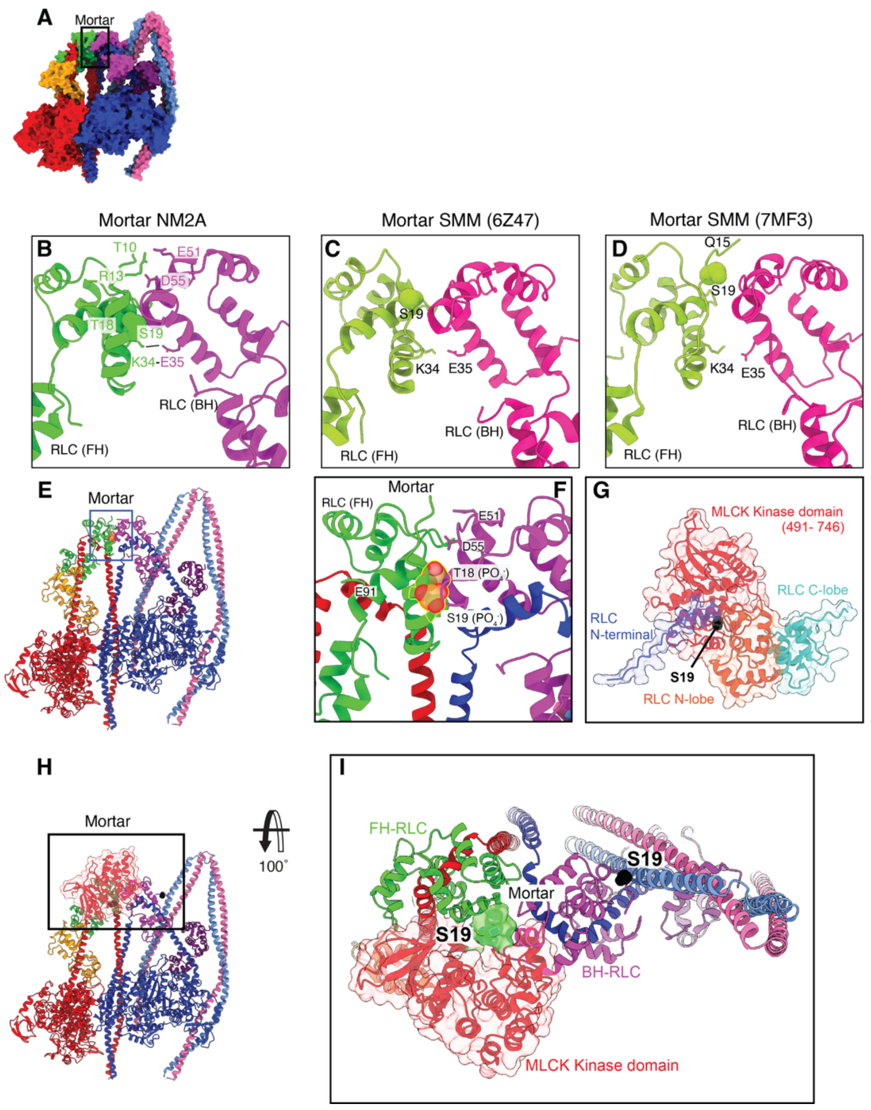

Fig. S4. Comparison of Mortar for NM2A and SMM and its phosphorylation by MLCK
(A) Colored surface view of the heads region (back view) to show the location of the FH RLC N-terminal region (Mortar: defined as residues 1-19 of the FH RLC). **(B)** Zoomed view of region boxed in **(A)** showing RLCs only (HCs omitted). Side chains of interacting residues between the two RLCs are shown. Side chains of the phosphorylatable S19 and T18 residues are shown as spheres. **(C, D).** Equivalent views (as in **B**) for SMM (6Z47 *(13)* and 7MF3 *(12)*.**(E)** Ribbon structure of the modelled phosphorylated molecule. **(F)** A zoomed in view of the phosphorylated mortar. Phospho-S19 and Phospho-T18 residues are shown as spheres, with side chains of clashing residues in the mortar interface shown as sticks. **(G)** The top scoring AlphaFold model of the MLCK Kinase domain (491-746) in complex with Bovine RLC (RLC_MLCK), showing the N-terminal extension of the RLC embedded in a groove of the kinase domain (S19 shown in black). **(H)** To place the kinase domain into the shutdown structure, the N-terminal region of the RLC kinase: RLC complex (residues 17 – 23) was aligned onto the RLC of the FH. (**I**) shows boxed region in **(H)** as a surface fill representation of the FH N-terminal extension (green) with the kinase domain (red). The kinase makes minimal contacts with the rest of the RLC.

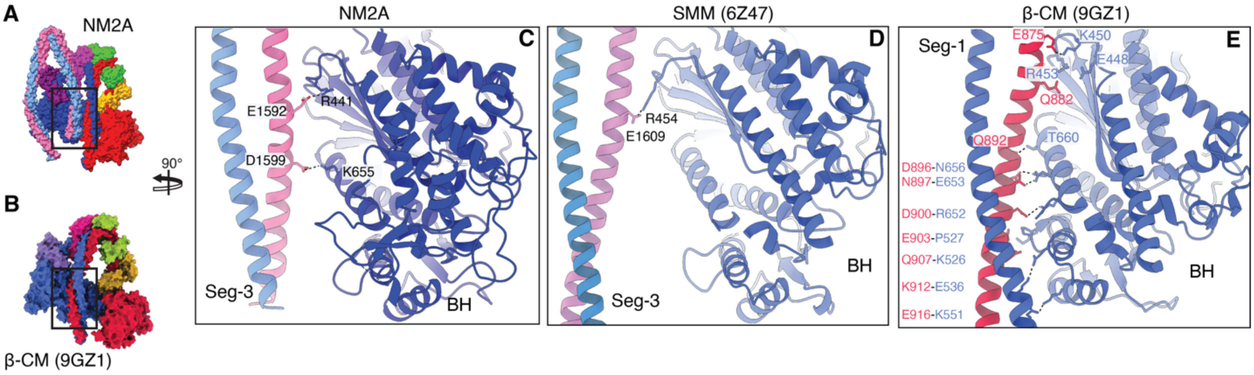

Fig. S5: Paths of Seg-1 and Seg-3 in β-CM and NM2A/SMM, respectively
(**A**) Colored surface view of the heads region of NM2A (face view) showing Seg-3 and its position close to the BH. (**B**) Colored surface view of the heads region of β-CM (face view: 9ZG1 *(22)* to show the location of Seg-1. (**C**) Zoomed and rotated view of the region of NM2A boxed in **A**. Side chain interactions between Seg-3 and the blocked head are shown. (Numbering excludes methionine as residue 1). No other interacting residues were present. (**D**) Equivalent view for SMM (6Z47 *(13)* as in **C**. (**E)** Zoomed and rotated view of the region of β-CM boxed in **B.** Side chains of interacting residues between Seg-1 and the mesa trail are shown (interacting residues are indicated on the LHS, as CC – BH). The view shown is the equivalent representation as in **C, D** for the alignment of β-CM (9GZ1) with NM2A and 6Z47, with Seg-1 occupying a similar space to Seg-3**.** The mesa trail in β-CM is extensive (*22*) and only some of the interactions reported are shown. Residues D896, N897, D900 and E903 (β-CM) are all in a region of acidic charge termed Ring-1 (see alignment in Fig. S6). We chose not to use 8ACT (*23*) for this comparison, as the sequence of β-CM is shorter (residues 1-842) and followed by a leucine zipper. In 9GZ1, the sequence of β-CM is longer (residues 1-1137), the leucine zipper is absent, and the curved conformation of S2 is closer to that observed in the thick filament structures.

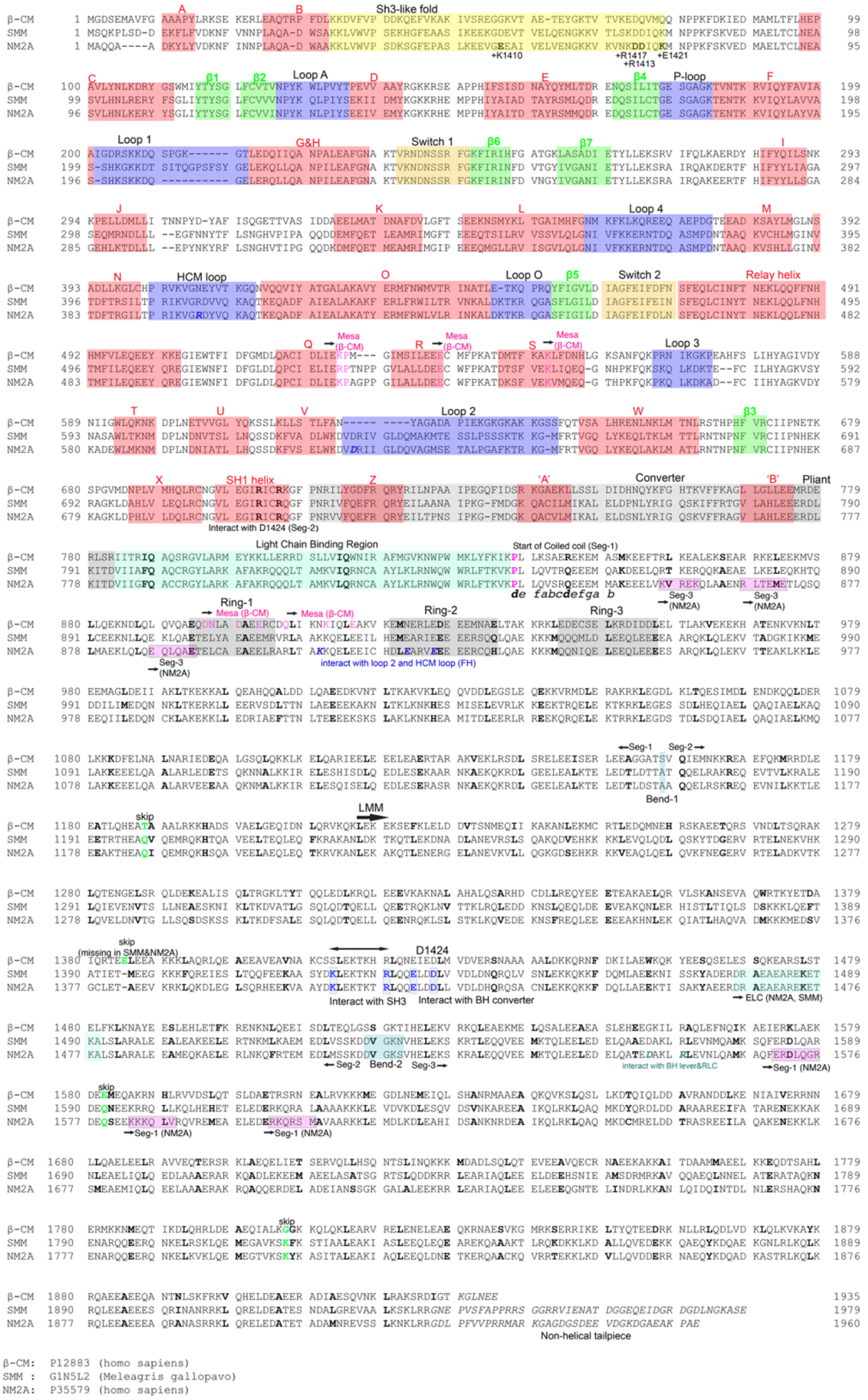

Fig. S6: Alignment of β-CM, SMM and NM2A heavy chains.
Sequences used are shown below the alignment. Regions within the motor domain, the light chain binding region and the coiled coil are annotated as shown. Bold residues from the invariant proline (magenta) are the ‘*d*’ residues of the coiled-coil heptad repeats. Positions of bends and ‘skip’ residues as indicated. Magenta ‘Mesa’ residues, indicate interacting residues in the mesa trail in β-CM HMM. Magenta boxed regions: interacting regions between Segs-1 and Seg-3 as shown in Fig. 3 for NM2A. Additional sites of interaction as indicated.

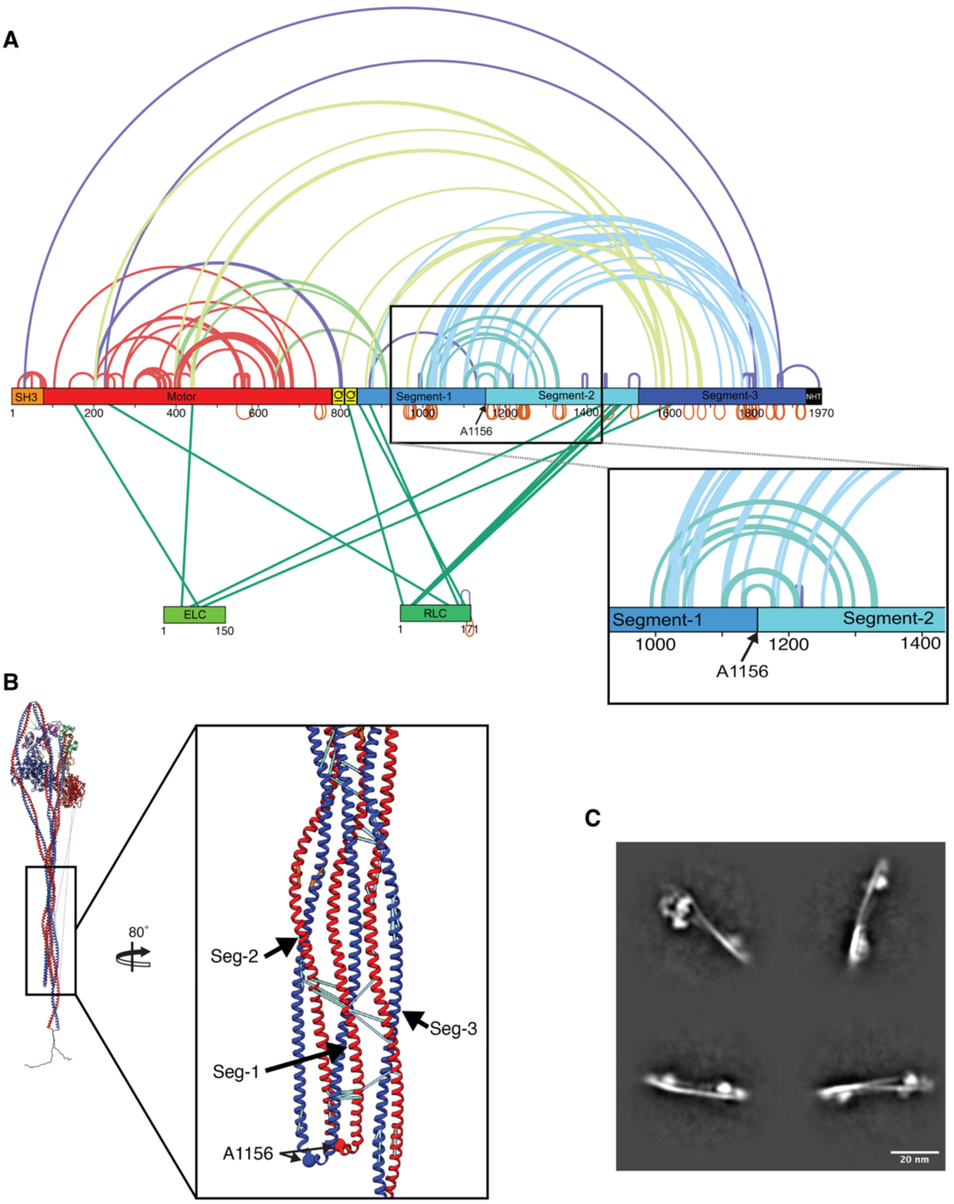

Fig. S7: XLMS data for shutdown NM2A.
(**A**) Linear diagram to show the domains of NM2A (SH3-like fold, motor etc). Crosslinked residues are indicated by the colored lines drawn. Colors show interactions within domains: red lines for cross-links within the motor, light blue lines between Seg-1 and Seg-3 of the coiled coil, blue-green between Seg-1 and Seg-2 of the coiled coil, light green lines between regions of the motor or coiled coil with light chains, light green for cross-links between motor and coiled coil, and purple for other cross-linked peptides. Orange cross-links signify self-linked peptides. (**B**) shows the shortest cross-link chain pair (under 27Å cut-off; colored according to (**A**) on the shutdown molecule for the boxed region to show the X-links between Seg-1, -2 and -3, placed on the full-length model structure for this region of the structure. **C**. 2D class averages of anti-parallel NM2A dimers (which encompass ~1% of the cryoEM dataset) that explain very long cross-links outside of the 27Å cut-off within the XLMS dataset.

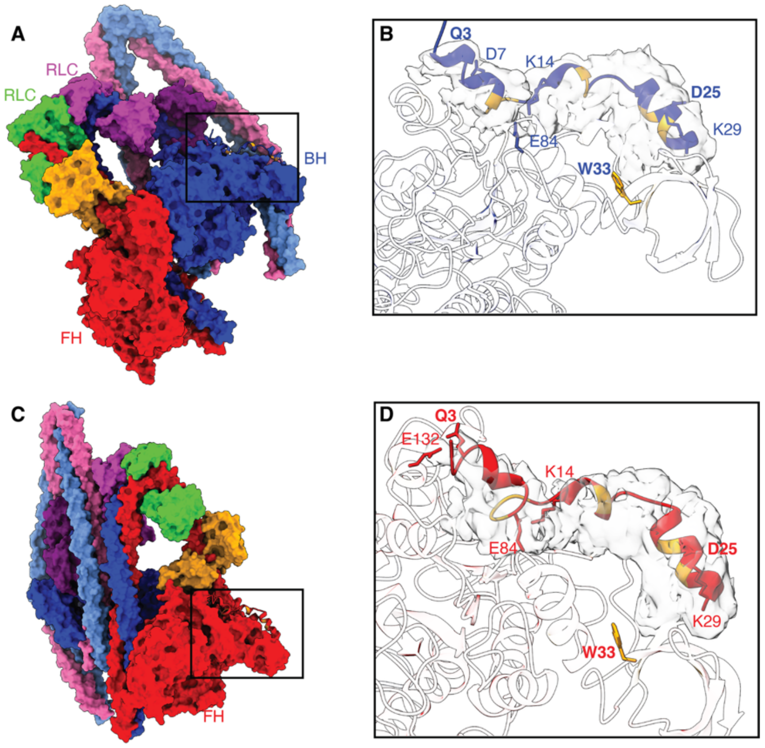

Fig. S8: N-terminal domains for Blocked Head and Free Head
(**A**) Colored surface view of the heads region to show the location of N-terminal domain of the BH heavy chain. (**B**) Zoomed view of region boxed in **A**. Side chains of interacting residues for the BH (Q3:D7, K14:E84,D25:K29) and FH (Q3:E132, K14:E84, D25:K29) are shown, with hydrophobic residues in this interface colored in orange. Q3, D25 and W33 are known sites of disease-causing mutations and are labelled in bold text. (**C, D**) Equivalent views as **A, B** for the N-terminal domain of the FH.

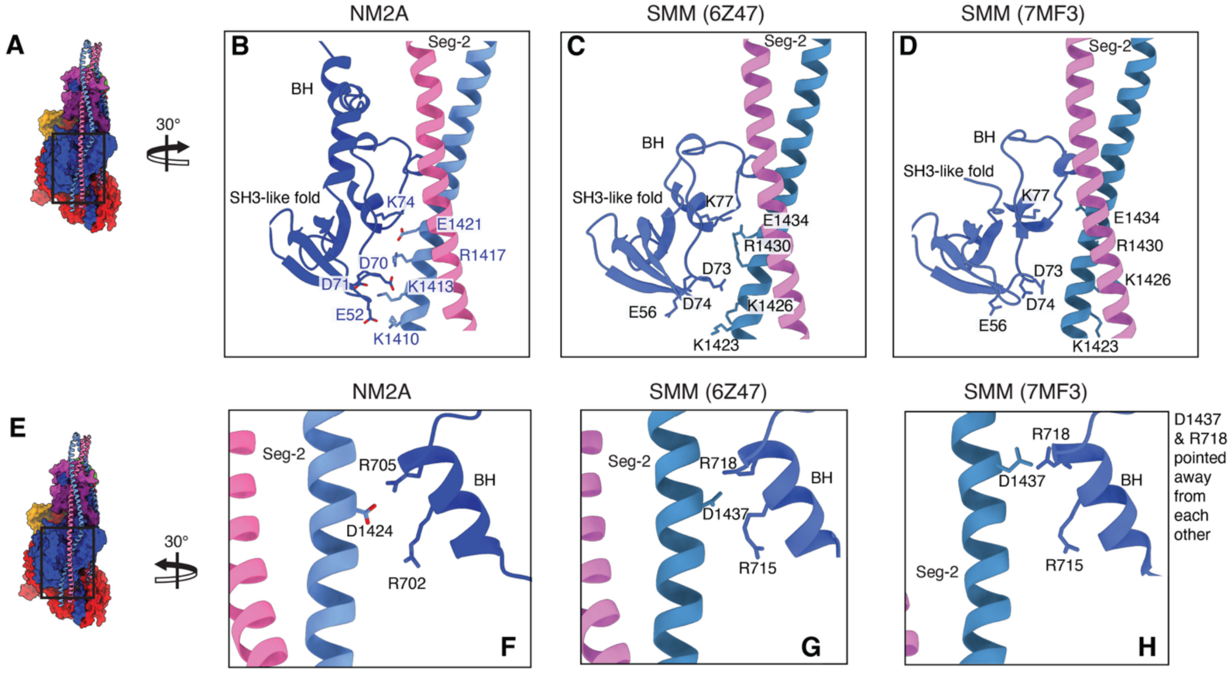

Fig. S9: **Comparison of Seg2-SH3 and Seg2-SH1-helix interactions in NM2A with SMM** (**A**) Colored surface view of the heads region (side view) to show the location of Seg-2 interacting with the groove formed within the BH heavy chain. (**B**) Rotated and zoomed view of region boxed in **A**. Side chains of interacting residues between the SH3-like fold and Seg-2 in the BH are shown. (**C, D**) Same representation as in **B** for SMM (PDB: 6Z47, (*1*)) and SMM (PDB:7MF3, (*2*)) structures (aligned with NM2A). (**F**) Rotated and zoomed view of region boxed in **E** for NM2A. Side chains of interacting residues between the SH1-helix and Seg-2 in the BH are shown. All three residues shown are known to have disease causing mutations. (**G, H**) Same representation as in **F** for 6Z47 and 7MF3 SMM structures**.**

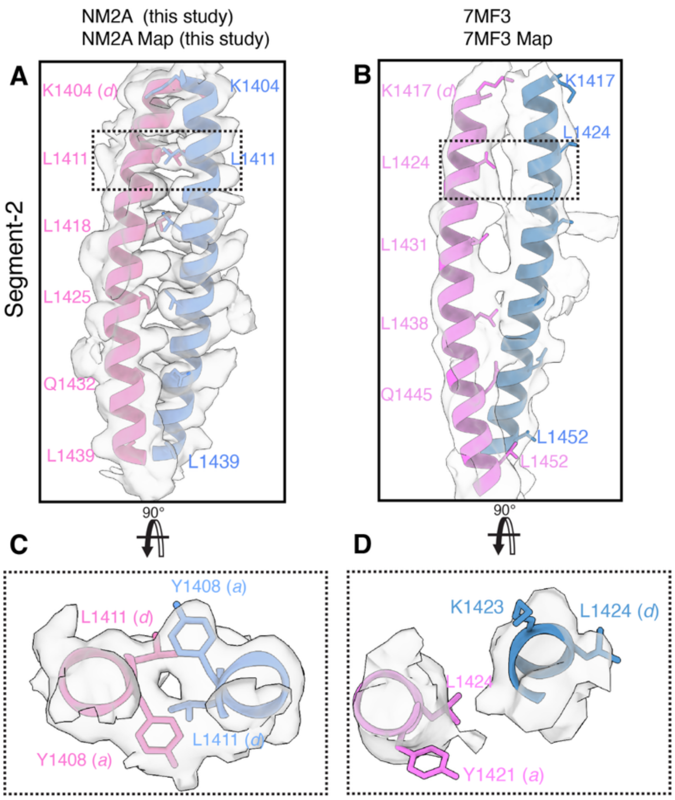

Fig. S10. Comparison of the coiled-coil models in Seg-2 of NM2A and SMM (7MF3)
(A) Representative 5 heptads of coiled coil in Seg-2 (chains G/H:1404-1439) of NM2A in the corresponding segmented cryo-EM map (zoned density displayed at a level of 0.02) showing side chains of *d* residues in the hydrophobic seam. (**B**) Equivalent representation of the corresponding section of Seg-2 in SMM (PDB:7MF3, (*2*)) in its respective map (EMD-23810, zoned density displayed at a level of 0.1). (**C**) Boxed region from (**A**) to show residues within the hydrophobic seam within the corresponding density map (zoned density displayed at a level of 0.1). The *a*-*a* and *d*-*d* pairing in the hydrophobic seam is visible. (**D**) Boxed region from **B** to show residues within the hydrophobic seam within corresponding density map (zoned density displayed at a level of 0.1) for 7MF3. Aberrant positions of *a* and *d* residues are visible.

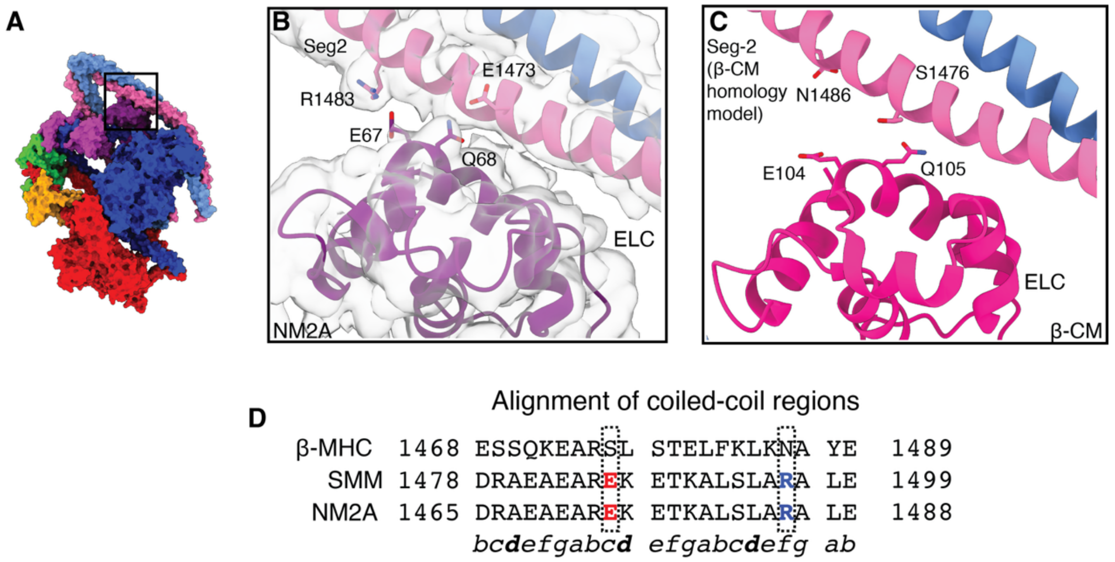

Fig. S11. Blocked Head ELC Seg-2 interactions
(**A**) Colored surface view of the heads region to show the location of BH-ELC Seg-2 interface. (**B**) Zoomed view of region boxed in A. Side chains of interacting residues are shown within the segmented map. (**C**) Equivalent representation of β-CM BH-ELC and a homology model for Seg-2 of β-CM, with corresponding β-CM BH-ELC residues shown. (**D**) Sequence alignment for human heavy chain sequences for β-CM, SMM, and NM2A for the interacting portion of Seg-2. The interacting residues are indicated in bold and color. The heptad position is shown below the alignment. Interacting residues in Seg-2 are not conserved in β-CM.

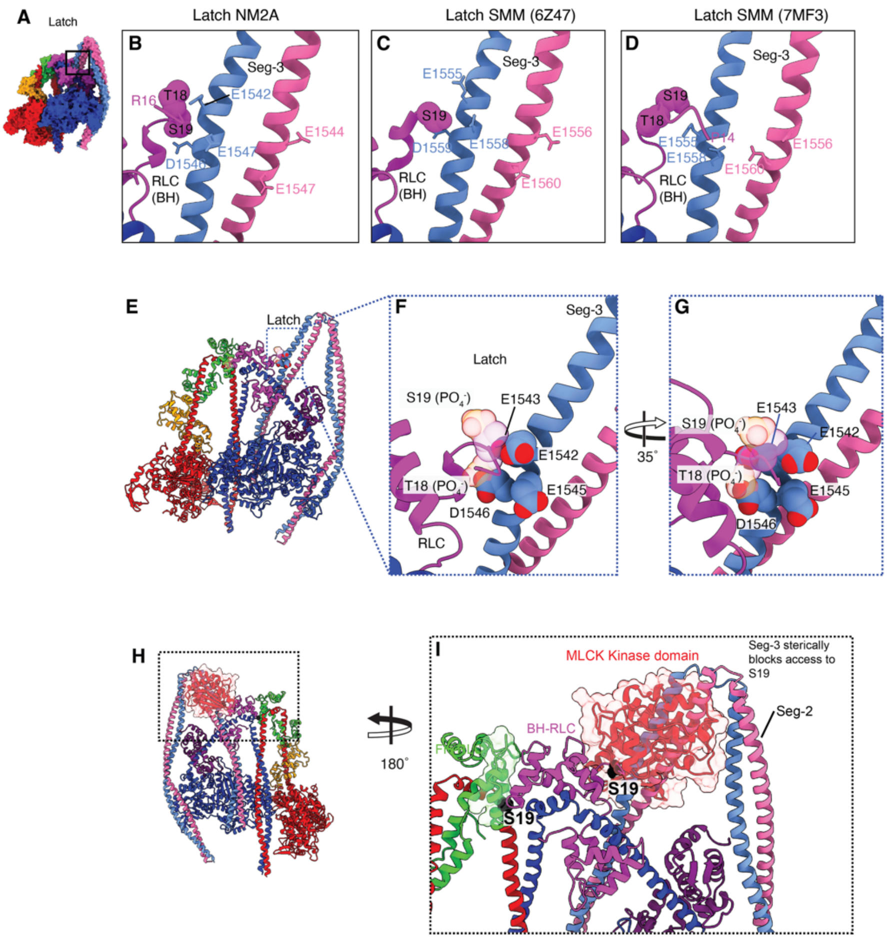

Fig. S12: Comparison of latch structures, effect of phosphorylation and MLCK kinase domain interaction.

(A) Colored surface view of the heads region (back view) to show the location of the BH RLC N-terminal region (Latch: defined as the N-terminal region of the BH RLC, residues 1-19). **(B)** Zoomed view of region boxed in **A.** Side chains of interacting residues between the BH-RLC and Seg-3 are shown. Side chains of the phosphorylatable S19 and T18 residues are shown as spheres. **(C, D).** Equivalent views as in **B** for 6Z47 and 7MF3. The side chains of negatively charged residues of Seg-3 for SMM are shown as sticks. **(E)** Ribbon structure of the modelled phosphorylated molecule showing the position of the latch (boxed region). (**F, G)** Zoomed in views of the phosphorylated latch. Phospho-S19 and Phospho-T18 residues are shown as spheres. Negatively charged residues in the local environment are shown. (**H**) The latch docked with the kinase domain of MLCK, in which the RLC of the kinase-RLC AlphaFold model is aligned as in Fig. S3. Boxed region in (**I**), shows how the kinase is sterically occluded by Seg-3 on the blocked-head and unable to interact with S19 in the latch.

Table S1. Microscope parameters

Microscope	Titan Krios
Magnification	96000
Voltage (kV)	300
Electron dose per image ($e^-/Å^2$)	36.94
Exposure time (s)	3.42
Number of fractions	50
Defocus range (mm)	-0.9 to -3.0 (0.3 steps)
Pixel size (Å)	0.82

Cryo-EM data collection statistics.

Supplementary Movies

Movie S1. Shutdown state of non-muscle myosin 2a (A) Cryo-EM density of NM2A (grey) shutdown heads region at an average resolution of 3 Å. Colored map representation showing heavy chain (blue, red), ELC (purple, orange), and RLC (magenta, green) in complex with segments 2 and 3 (light blue, pink) and fitted pseudo-atomic model. (B) Closeup view of the head tail junction. (C) Close up view of the free-head RLC mortar (dark green) with potentially interacting residues (sticks) in both RLCs shown. (D) MLCK kinase domain docked onto the S19 of the FH RLC showing few steric clashes with the free head.

Movie S2. **Three principal components from 3D variability analysis of NM2A.** The movie shows conformational heterogeneity of NM2A revealed by 3D variability analysis in CryoSPARC. Principal components 1–3 (PC1–PC3) capture the dominant continuous motions within the dataset. Each component is displayed sequentially in series between the two extreme conformations along that component, highlighting the major structural changes within the molecule.

Movie S3. **Tail Flexibility.** The movie shows two-dimensional class averages of non-muscle myosin-2A (NM2A) molecules, arranged to illustrate the range of conformations reflecting in-plane flexing of the tail region. Scale bar, 20 nm.
